# Evidence of altered DNA integrity in the brain regions of suicidal victims of Bipolar Depression

**DOI:** 10.4103/0019-5545.70974

**Published:** 2010

**Authors:** Mohammed S. Mustak, Muralidhar L. Hegde, Athira Dinesh, Gabrielle B. Britton, Ruben Berrocal, K. Subba Rao, N. M. Shamasundar, K. S. J Rao, T. S. Sathyanarayana Rao

**Affiliations:** 1 Department of Biochemistry and Nutrition, Central Food Technological Research Institute, Mysore, India; 2 Department of Applied Zoology (Presently), Mangalore University, Mangalagangothri, India; 3 Department of Biochemistry and Molecular Biology (Presently), University of Texas Medical Branch, Galveston, Texas, USA; 4 Malankara Orthodox Syrian Church Medical College and Hospital, Kolencherry, India; 5 Institute for Scientific Research and Technology Services (INDICASAT), Panama, India; 6 National Secretariat of Science, Technology and Innovation (SENACYT), City of Knowledge, Panama, India; 7 Jawaharlal Nehru Technological University, Hyderabad, India; 8 Department of Anatomy, JSS Medical College Hospital, M.G. Road, Mysore, India; 9 Department of Psychiatry, JSS Medical College Hospital, M.G. Road, Mysore, India

**Keywords:** Bipolar depression, DNA fragmentation, DNA conformation, strand breaks, DNA stability, brain regions, trace metals, oxidative stress

## Abstract

Deoxyribonucleic acid (DNA) integrity plays a significant role in cell function. There are limited studies with regard to the role of DNA damage in bipolar affective disorder (BP). In the present study, we have assessed DNA integrity, conformation, and stability in the brain region of bipolar depression (BD) patients (n=10) compared to age-matched controls (n=8). Genomic DNA was isolated from 10 postmortem BD patients’ brain regions (frontal cortex, Pons, medulla, thalamus, cerebellum, hypothalamus, Parietal, temporal, occipital lobe, and hippocampus) and from the age-matched control subjects. DNA from the frontal cortex, pons, medulla, and thalamus showed significantly higher number of strand breaks in BD (*P*<0.01) compared to the age-matched controls. However, DNA from the hippocampus region was intact and did not show any strand breaks. The stability studies also indicated that the melting temperature and ethidium bromide binding pattern were altered in the DNA of BD patients’ brain regions, except in the hippocampus. The conformation studies showed B-A or secondary B-DNA conformation (instead of the normal B-DNA) in BD patients’ brain regions, with the exception of the hippocampus. The levels of redox metals such as Copper (Cu) and Iron (Fe) were significantly elevated in the brain regions of the sufferers of BD, while the Zinc (Zn) level was decreased. In the hippocampus, there was no change in the Fe or Cu levels, whereas, the Zn level was elevated. There was a clear correlation between Cu and Fe levels versus strand breaks in the brain regions of the BD. To date, as far as we are aware, this is a new comprehensive database on stability and conformations of DNA in different brain regions of patients affected with BD. The biological significance of these findings is discussed here.

## INTRODUCTION

Bipolar depression (BD) is one of the major psychiatric disorders characterized by recurrent depressive and manic episodes.[1] BD affects about 1% of the population and causes severe neuropsychological impairments. The illness is implicated in functional impairment and represents an important risk factor for suicidal behavior.[2] Twin, adoption and family studies, show that genetic factors also contribute to the etiology of this disorder.[1] More recently, remarkable progress has been made in identifying the changes in the brain, related to the pathophysiology of BD. The neurochemical and brain imaging studies have demonstrated volume loss in the brain, in BD.[3,4] Studies have also shown a reduction in the numerical density of neurons in several brain regions including the anterior cingulate cortex and the hippocampus of subjects with BD.[5,6] The above-mentioned studies indicate that cell death plays a significant role in BD. Furthermore, the studies suggest that oxidative stress plays a role in the etiology of BD.[7–12]

The apoptotic cell death of neurons is hypothesized to have a role in neuropsychiatric disorders.[13–15] There are limited studies indicating the presence of DNA damage in BD.[11,15–19] However, DNA fragmentation has also been reported with neurodegenerative disorders.[20–23] The aim of the current study is to assess the genomic integrity in terms of DNA fragmentation, conformation, and stability in the different brain regions of BD and to ascertain whether altered genome integrity plays a role in thepathophysiology of BD.

## MATERIALS AND METHODS

### Materials

Radiolabeled^3^[H]-TTP (Sp.Act.40Ci/nmol) was purchased from Amersham Radiochemicals, UK. Ribonuclease A (RNAse A), Proteinase k, Deoxyribonuclease I (DNAse I), dATP, dTTP, dCTP, dGTP, low melting agarose, DNA polymerase I (from *E. coli*), terminal deoxynucleotidyl transferase enzymes, 1 kb and 100 bp DNA ladders, and lamda DNA ladder were purchased from Genei, India. Ethidium bromide (EtBr), Hepes, and Tris buffers were purchased from Sigma Chemicals (USA). All other chemicals were of analytical grade and were purchased from Sisco Research Laboratories, Mumbai, India.

### Brain tissues

Eight normal and ten BD postmortem human brain samples were collected from the Depression Brain Bank of the JSS Medical College and Hospital, Mysore, India. Autopsies were performed on both sets with written informed consent obtained from the direct next of kin. The control human brains were collected from accident victims, who had no history of long-term illness, psychiatric diseases, dementia, or any neurological disease, prior to death. The BD brains were postmortem specimens and were diagnosed by using postmortem psychiatric analysis by structural clinical interviews and by referring to their medical records by psychiatrists. We found that the study subjects were not on any psychotropic medication prior to or at the time of death. In the present study, the brains were obtained from the BD suicidal victims, who died by hanging/ burns only and not by consuming poison. We have excluded subjects who had drug and alcohol abuse. The average postmortem interval between the time of death and collection of brain and freezing was ≤ six hours. The brain tissue was isolated and stored frozen at -80°C till the analysis. The clinical characteristics and agonal states of eight healthy controls and ten subjects with BD matched for age, postmortem interval (PMI), pH, sex, and freezer storage time, are provided in Table 1.

**Table 1:** Demographic data of healthy control and BD subjects

Number	Age, year	PMI, hour	Tissue pH	Sex	Cause of death
Controls subjects					
N-1	25	4	6.76	F	Road traffic accident
N-2	38	5	6.61	F	Snake bite
N-3	21	4	6.71	F	Accidental burns
N-4	18	4	6.45	M	Road traffic accident
N-5	22	5	6.68	M	Road traffic accident
N-6	24	6	6.72	M	Road traffic accident
N-7	30	3	6.54	F	Accidental burns
N-8	37	8	6.45	M	Fall from height
Mean±SD	26±7.4	4.9±1.6			
Bipolar-depression					
BD-1	25	4	6.67	M	Suicide by hanging
BD-2	20	5	6.23	F	Suicide by burning
BD-3	19	4	6.70	M	Suicide by hanging
BD-4	18	3	6.51	M	Suicide by hanging
BD-5	20	4	6.31	F	Suicide by hanging
BD-6	21	4	6.53	F	Suicide by burning
BD-7	35	4	6.32	M	Suicide by hanging
BD-8	23	6	6.27	M	Suicide by hanging
BD-9	21	4	6.40	F	Suicide by hanging
BD-10	30	5	6.35	F	Suicide by burning
Mean±SD	23.2±5.4	4.4±1.1				

N, normal; BD, bipolar disorder; PMI, postmortem interval; SD, standard deviation

### Isolation of DNA from brain tissue

Genomic DNA was isolated from ten regions (Parietal, temporal, and occipital lobes, hippocampus, thalamus, cerebellum, hypothalamus, medulla, pons, and frontal cortex) of the frozen brain tissue by the standard ’phenol-chloroform extraction’ method of Sambrook *et al*.,[24] with some modifications, to prevent DNA fragmentation during isolation. The concentration of DNA was measured using the UV/ Visible spectrophotometer and noting the absorbance at 260 mm, and the purity was checked by recording the ratio of absorbance at 260 nm/ 280 nm, which should be ideally between 1.6 and 1.8.

- DNA stability
  - Agarose gel electrophoresis
    The integrity or damage in genomic DNA was assessed by running neutral and alkaline gel electrophoresis. The migration pattern in neutral gels reflected the double strand breaks present in the DNA, and the migration pattern in alkaline gels showed both single and double strand breaks.[25] The neutral gels were electrophoresed on 1.8% agarose gels in Tris-acetate-EDTA buffer (pH 8.0) at 4 V/cm for four hours and stained with EtBr for 15 minutes. Three micrograms of DNA was loaded in each well. DNA ladders (1 kb and 100 bp) were used as molecular weight markers. The stained gels were analyzed in the UV gel documentation system.
  - Single strand breaks:
    Single strand breaks (SSBs) are calculated through the incorporation of^3^[H]-TMP into the DNA samples, when incubated with *E. coli* DNA polymerase I (Klenow Fragment) in a nick translation assay. DNA polymerase I adds nucleotides at the 3’-OH end of an SSB, generated by various means, using the other strand as the template. When one of the deoxynucleotide triphosphates is labeled, then the incorporation of radioactivity into the substrate DNA will be proportional to the number of SSBs present in the DNA sample. During the standardization of the assay conditions with the plasmid DNA (Cos T fragment of ג phage) having a known number of SSBs, it has been found that an average of 1500 nucleotides are added at each of the 3’-OH groups. From this, it is inferred that each picomole of TMP incorporated is equivalent to 1.6 × 10^9^3’-OH groups or SSBs. In a total reaction volume of 50 *μ*l, the assay mixture consisted of: 40 mM Tris-HC1, pH 8.0, 1 mM β-mercaptoethanol, 7.5 mM MgCl_2_, 4 mM ATP, 100 *μ*M each of dATP, dCTP, and dGTP, and 25 *μ*M of dTTP, 1 *μ*Ci of ^3^[H]-TTP, and 1 *μ*g of genomic DNA and 1 U of E.coli DNA polymerase I.
  - Double strand breaks (DSBs):
    Terminal deoxynucleotidyl transferase catalyzes the addition of deoxynucleotides to the 3’ termini of DNA and does not need direction from the template strand. Here, the 3’-ends of the duplex DNA act as substrates. The incorporation of^3^[H]-dTTP into the DNA will be proportional to the number of DSBs in the DNA. From the conditions and incubation[26–27], it is assumed that about 50 TMP residues are added at each of the 3’-ends of the duplex DNA. From this, it is calculated that each femtomole of TMP incorporation will be equivalent to 1.2 × 10^7^3’-ends or half of that number minus one DSB. The assay mixture for the terminal transferase reaction consisted of a total volume of 50 *μ*1 : 100 mM sodium cacodylate buffer, pH 7.0, 1 mM CoCl_2_, 0.2 mM DTT, 1 *μ* Ci of^3^[H]-dTTP, 1 *μ*g DNA, and 1 U of the enzyme.
  - Melting temperature (t_m_) of the genomic DNA
    To determine the physical state of the DNA in BD depression brain, the melting profiles of DNA isolated from 10 different brain regions of BD depression and age-matched control groups were examined. DNA was dissolved in 0.01M Hepes buffer, pH 7.4. The DNA was used at a concentration of 20 *μ*g/mL. The melting profiles (t_m_) of DNA from the control and BD depression were recorded on a spectrophotometer, equipped with a thermo programer and data processor (Amersham, Hong Kong). The hyperchromicity changes of the DNA were recorded from 20 to 95°C with 1°C increment/ minute. The temperature point at which there was a 50% hyperchromic shift was taken as t_m_of the DNA sample. The t_m_values were determined graphically from the hyperchromicity versus temperature plots. The precision in the t_m_values were estimated in triplicate with ± 0.05°C deviation.
  - Ethidium bromide (EtBr) binding studies
    The quantification of EtBr bound in moles per base pair of genomic DNA was measured in 0.01 M Hepes, pH 7.4[28], using HITACHI F-2000 Fluorescence Spectrophotometer. The fluorescence was measured using a constant amount of DNA [2 *μ*g] with increasing EtBr [1 *μ*g to 2 *μ*g] against the blank containing no DNA. The measurements were performed keeping excitation at 535 and emission at 600 nm with 10 mm path-length. The maximum amount of EtBr bound per base pair of DNA was calculated using Scatchard plots of ‘r’ versus ‘r/Cf’.
- DNA conformation:
  Circular Dichroism (CD) studies
  The CD spectra (190-330 nm) were recorded for genomic DNA in 0.01 M Hepes buffer (pH 7.4) on a JASCO-J720 Spectropolarimeter, using 1 mm path length. Each spectrum was the average of quadruplicate recordings. Twenty-five micrograms of DNA from each sample was used. The DNA conformations were characterized from the CD spectra using the reference of Gray *et al*.[29]
- Trace elemental analysis:
  Trace metal analysis was done by inductively coupled plasma atomic emission spectroscopy (ICP-AES), as described previously.[38]
- Statistical analysis
  All the data obtained in this study were statistically treated and the significance of the differences between the control and BD depression were calculated according to the Student’s *t* test. The statistical analysis was carried out by using the Microsoft Excel 2000 Software.

## RESULTS

The brain samples from BD patients and controls were matched as precisely as possible in postmortem interval, age, tissue pH, and freezing storage time [Table 1]. The representative anatomical areas had been dissected under cold conditions and transported on dry ice and stored at –80°C. The DNA was isolated from 10 different regions namely, parietal, temporal, occipital lobes, hippocampus, thalamus, cerebellum, hypothalamus, medulla, pons, and frontal cortex from eight control and ten BD brains.

### Agarose gel electrophoresis

**Figure 1:**
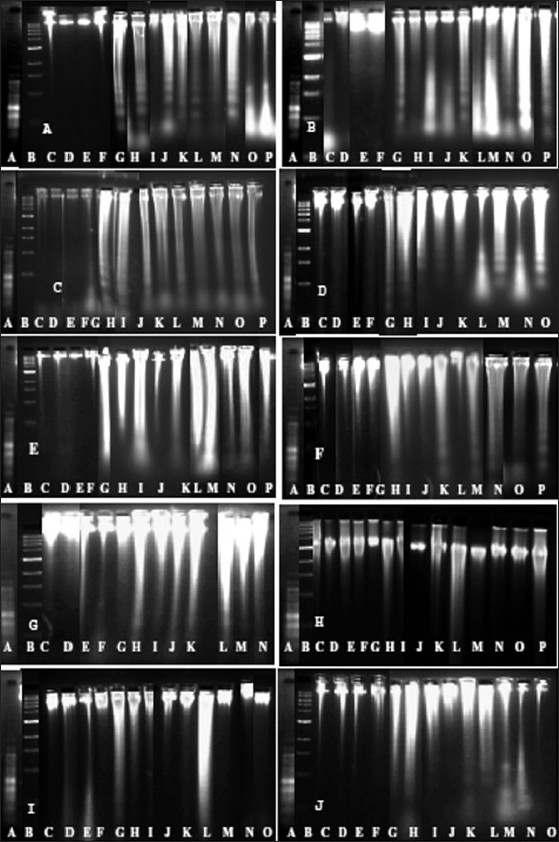
Neutral agarose gel electrophoresis pattern of genomic DNA isolated from BD vs. age-matched control brains. Neutral gels were run on 1.8% agarose. 2 μg of each DNA sample was used for gel studies. Lanes A and B in each gel represent the 100 bp ladder and 1 kb DNA marker, respectively. Figure 1A represents the DNA from the pons region where lanes C to F are the control subjects’ DNA and lanes from G to P are BD depressive brain DNA. Figure 1B represents the DNA isolated from the medulla region where lanes C to D represent the control subjects’ DNA and lanes G to P the BD-DNA. Figure 1C represents temporal lobe DNA where lanes C to F are control DNA and lanes G to P are BDDNA. Figure 1D represents the thalamus DNA where lanes C to F are control DNA and lanes from G to O are from the BD-DNA. Figure 1E represents the cerebellum where lanes C to F are from the control DNA and lanes G to P are from the BD-DNA. Figure 1F represents the frontal cortex DNA where lanes C to F are from control DNA; lanes G to O are the fragmented DNA from BD. Figure 1G represents the parietal lobe DNA where lane C to F are control DNA and lane G to N are from BD DNA. The Figure 1H represents DNA from the hippocampus region where lanes C to F are control DNA and lanes from G to O are BD-DNA. The Figure1I represents the DNA from the hypothalamus where lanes C to F are control DNA and lanes G to O are from the BD-DNA., Figure1J represents the DNA from the occipital lobe where lanes C to F are from the control DNA and lanes G to O are from the BD-DNA.

The measurements of DNA fragmentation or damage were performed in 10 different brain regions of BD and age-matched control subjects by agarose gel electrophoresis. The areas like Pons, medulla, temporal lobe, thalamus, cerebellum, and frontal cortex region of the BD brains showed a higher level of DNA fragmentation when compared to the age-matched control brains [Figure 1 A–F]. However, moderate DNA damage was observed in the parietal lobe, occipital lobe, and hypothalamus of BD brains compared to controls [Figure 1 F–J]. Interestingly, the hippocampus of both BD and age-matched control brains showed no DNA fragmentation, and the DNA was intact [Figure 1H].

### Single Strand Breaks

**Figure 2:**
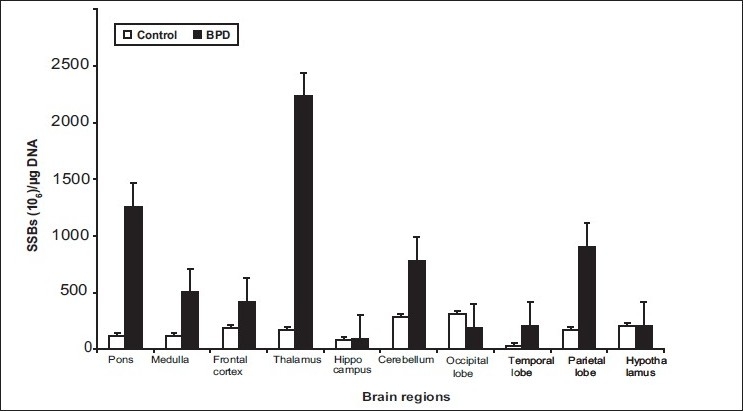
Assessment of single strand breaks (SSBs) in DNA isolated from 10 different regions in the brains of BDdepression and control subjects. The SSBs in DNA were determined through nick translation type of incubation with E.coli DNA-polymerase I. The bar chart represents SSBs values mean±SD for eight control and ten BD-depression brain DNA samples.

Single Strand Breaks are predominantly seen during DNA damage in cells. Single-stranded breakage is the end point of several types of structural insults inflicted on the genome by both endogenous and exogenous agents.[30] Figure 2 shows the number of SSBs per microgram of genomic DNA, isolated from various brain regions. Accumulations of SSBs were more frequent in BD than in healthy controls in the following regions. Thalamus (*P*<0.001), pons (*P*<0.01), cerebellum (*P*<0.05), parietal lobe (*P*<0.02), and frontal cortex (*P*<0.05) accumulated significantly higher number of SSBs compared to healthy controls, from their respective regions. [Figure 2]. Interestingly, there were negligible numbers of SSBs in the hippocampus region of BD brains as well as controls.

### Double Strand breaks

**Figure 3:**
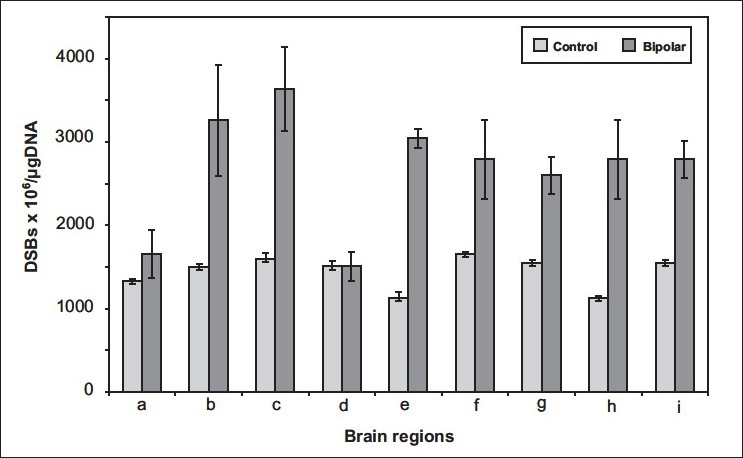
Assessment of double strand breaks (DSBs) in DNA isolated from 10 different regions of BD depression and control brains. (a) Hypothalamus, (b) Frontal cortex, (c) Thalamus, (d) Hippocampus, (e) Cerebellum, (f) Temporal lobe, (g) Pons, (h) Medulla, (i) Parietal lobe. DNA was measured using the terminal transferase assay. The bar-chart represents the mean of DSBs values±SD for eight controls and ten BD brain DNA samples.

The DNA isolated from the thalamus, frontal cortex, cerebellum, pons, and temporal lobe showed significantly higher number of DSBs than the respective controls (P<0.01) [Figure 3]. Hippocampus showed no DSBs in both the control and BD samples. Regions such as thalamus, pons, medulla, cerebellum, and parietal lobe accumulated both SSBs and DSBs, more in BD than in controls, whereas, the frontal cortex, accumulated more DSBs than SSBs. We also observed less number of DSBs in the hypothalamus DNA. These results showed that frontal cortex had more DSBs than SSBs, whereas, the thalamus, cerebellum, parietal lobe, and pons had a presence of both accumulated DSBs and SSBs. Aninteresting finding was that the hippocampus region showed the least accumulation of SSBs and DSBs in both control subjects and BD human brain samples.

### Trace elemental analysis:

Trace metal data provided new information on genomic instability in the frontal cortex, while no change was observed in the hippocampus. Redox metals like Cu and Fe were elevated in the depressive brain frontal cortex, where the SSBs were also elevated. In addition, the antioxidant Zn was reduced. These data provide direct evidence on the role of redox metal imbalance in DNA instability. This is the first finding in the literature. We did not observe DNA instability in the hippocampus. In support of this, we could find no changes in Cu, Fe, and Zn levels. This data too further confirms the role of metal imbalance in DNA instability and neuronal dysfunction [Table 2].

**Table 2:** Trace metal concentrations in two regions of human depressive and control brains.

Brain regions	Trace metals	Control subjects (N=10)	Depressive (N=10)
Frontal cortex	Cu	4.0±1.7	8.0±1.5*
	Al	58 0±20	113±30*
	Fe	50.8±13.6	75±15.5*
	Zn	7.5±0.5	4.5±0.4**
Hippocampus	Cu	4.0±1.0	4.5±1.5
	Al	45±16	47±14
	Fe	26.6±7.6	24.6±8
	Zn	6.5±0.5	5.5±0.2

(Concentration in mg/g of wet weight of tissue). Mean±SD of ten brains

### Melting Temperature studies

The t_m_ and hyperchromicity values of genomic DNA dissolved in 0.01 M Hepes buffer, pH 7.4, is shown in Table 3. The data showed that the t_m_ was significantly low for genomic DNA isolated from the BD brain region. The genomic DNA from the temporal lobe (*P*<0.001), medulla (*P*<0.001), frontal cortex (*P*<0.001), thalamus (*P*<0.001), pons (*P*<0.05), and cerebellum (*P*<0.05) of BD brain were significantly unstable compared to the age-matched controls. Consistent with DNA damage data, no significant difference was observed in t_m_ for DNA isolated from the hippocampus and hypothalamus of both control and BD brains. The decrease in the t_m_ of DNA isolated from the thalamus, temporal lobe, medulla, frontal cortex, cerebellum, and pons might be due to more strand breaks and the resulting destabilization of DNA.

**Table 3:** Melting temperature (t_m_) of DNA isolated from control and BD-affected postmortem human brains (Mean±SD)

Brain regions	Control	BD-depression
Thalamus	76.6±7.5*	61.34±6.25
Frontal cortex	81.6±3.5*	64.7±7.5
Medulla	74.17±10.1**	60.8±4.7
Temporal lobe	78.5±7.1*	59.8±2.7
Cerebellum	58.9±1.01**	55.8±3.8
Pons	73.8±8.2*	69±5.4
Occipital lobe	68±5	61.2±4.4
Hypothalamus	69±3.1	70.1±5
Hippocampus	61.9±2.6	61.5±1.49

Values are as degrees centigrade. All the values are means ±SD of eight control and ten BD samples. The melting temperature (t_m_) was calculated as the point of 50% hyperchromic shift. The statistical significance was calculated using Student’s *t* test.

* These values are significantly different from the corresponding control values at *P*<0.001.

** These values are significantly different from the corresponding control values at *P*<0.02.

### EtBr binding studies

**Figure 4:**
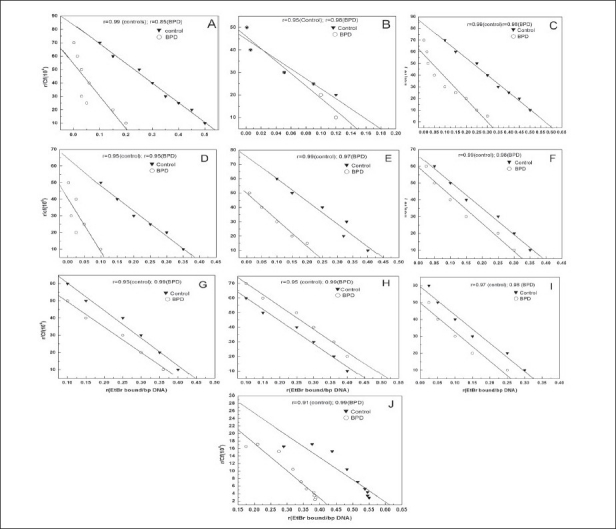
Scatchard plot of EtBr binding to DNA isolated from human BD depressive and control subjects. (A) Cerebellum, (B) Hippocampus, (C) Medulla, (D) Pons, (E) Frontal cortex, (F) Temporal lobe, (G) Occipital lobe, (H) Parietal lobe, (I) Hypothalamus, (J) Thalamus. Increasing amount of EtBr was added to a fixed concentration of DNA in 1 mL reaction mixture (0.01 M Hepes, pH 7.4). Fluorescent measurements were taken at RT setting, with excitation at 535 nm and emission at 600 nm. The Scatchard plot was drawn using the least square method. The same analysis was carried out for all the DNA samples extracted from the ten regions of eight control and ten BD-depression brain samples

The number of EtBr molecules bound per base pair (bp) of DNA is represented in Table 4. Scatchard plots of ten regions from the eight controls and ten BD brains DNA are shown in Figure 4. The EtBr binding data show that the DNA isolated from the thalamus, medulla, pons, cerebellum, temporal lobe, and frontal cortex have significantly less EtBr/ bp in BD compared to the age-matched controls (*P*<0.02). However, the EtBr bound/ bp to DNA from the hippocampus, occipital lobe, and hypothalamus does not differ significantly between BD and control subjects.

**Table 4:** Scatchard plot data on Ethidium Bromide binding pattern

Brain regions	Control	BD
Thalamus	0.63±0.09	0.44±0.07*
Medulla	0.61±0.11	0.32±0.15*
Pons	0.38±0.1	0.12±0.03*
Cerebellum	0.53±0.16	0.20±0.04*
Hippocampus	0.18±0.06	0.15±0.1
Temporal lobe	0.39±0.08	0.32±0.03*
Occipital lobe	0.45±0.12	0.40±0.08
Parietal lobe	0.45±0.15	0.51±0.11
Hypothalamus	0.32±0.08	0.26±0.13
Frontal cortex	0.44±0.1	0.25±0.03*

Bound Ethidium Bromide (EtBr) per base pair (bp) of DNA from BD and control samples were calculated using Scatchard plots. Values are as mean of number of EtBr molecules bound per base pair of DNA±SD. The values with asterisk

(*) are significantly different from the corresponding control values at *P*<0.02 of eight control and ten BD DNA samples. The statistical significance was calculated using Student’s *t* test.

### CD studies

**Figure 5:**
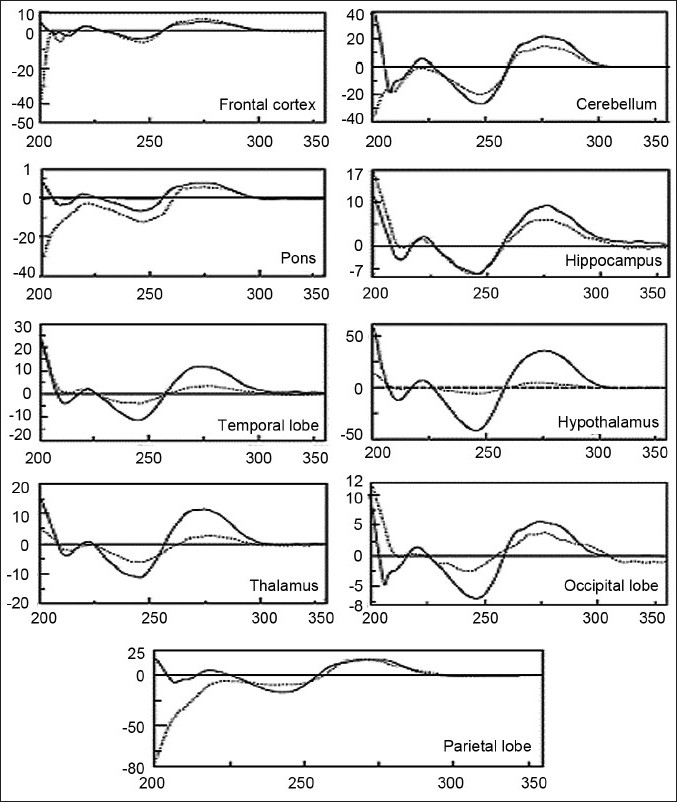
Circular Dichroism (CD) spectra of DNA isolated from frontal cortex, cerebellum, pons, parietal lobe, temporal lobe, hypothalamus, thalamus, occipital lobe, and hippocampus. Solid line represents control DNA and dotted line represents BD DNA. The recordings were performed in 0.01 M Hepes buffer, pH 7.4. Each spectrum was representative of the average of three recordings. CD was performed on eight control brains and ten BD depressive brain DNA samples; and one spectrum from each group has been represented.

The CD spectra of DNA isolated from the frontal cortex, cerebellum, pons, thalamus, temporal lobe, and parietal lobe differ from their respective controls [Figure 5]. The negative peak at 200 nm was observed more in the frontal cortex, cerebellum, pons, and parietal lobe of the BD brain compared to the age-matched control subjects. The presence of a negative peak at 200 nm is the characteristic feature of secondary (altered) B-DNA. Furthermore, the positive peak at 275 nm and negative peak at 245 nm were consistently low for the DNA isolated from the temporal lobe and thalamus of the BD brain rather than for the age-matched control subjects. The CD spectra of the BD-thalamus and pons showed the blue shift compared to the controls. These results showed for the first time that genomic DNA isolated from the BD patients’ brain regions had altered conformation.

## DISCUSSION

DNA conformation plays a significant role in cell physiology. The present study is focused in understanding the DNA conformation and integrity in brain regions of BD in comparison with age-matched control subjects. The changes include DNA damage in the form of SSBs and DSBs and DNA instability as indicated by the t_m_ and EtBr binding studies. Interestingly, the changes observed appear to be brain region-specific. The DNA isolated from the thalamus, frontal cortex, medulla, cerebellum, temporal lobe, and pons are largely affected, while the parietal lobe and hypothalamus are less affected. However, the hippocampus region does not show any DNA fragmentation both in the control and BD brains. Previous MRI studies have indicated volume reductions in different regions of the BD brain compared to the controls.[3,31,33–35] The biochemical factors, namely, mitochondrial DNA-deletion andapoptosis, are associated with the etiology of BD.[9–12,36–38]

The CD studies showed that DNA extracted from the frontal cortex, cerebellum, pons, and parietal lobe of the BD depressive brain were in the B-A mixed or altered conformation. However, both control and BD hippocampus DNA were shown to be in the intact, normal, right-handed helical B-DNA conformation. It was previously shown that the genomic DNA extracted from severe Alzheimer’s disease (AD) hippocampus was predominantly in the Z-DNA conformation rather than the usual B-DNA conformation,[23] and it was proposed that the change in conformation of DNA altered the chromatin integrity, and thus could alter DNA transactions and in turn disrupt cell functioning. It was earlier shown that DNA fragmentation reduced the high activation energy barrier required to induce the conformational and topological changes in DNA.[23] In addition, astudy showed that there was an empiric link between late-life depression and AD, suggesting that depression may lead to the development of AD in some individuals.[39]

Our study is new and shows that there is a selective increase of SSBs and DSBs in DNA in the thalamus, pons, frontal cortex, and medulla of BD-depressive brains compared to the age-matched controls. In contrast to a previous study that reported decreased DNA fragmentation in anterior cingulate cortex?[17] the present study shows that the hippocampus DNA is not affected in terms of SSBs and DSBs in both control and BD-depressive brains. Consistent with the current finding, Benes *et al*.[17] shows that no apoptosis is observed in the hippocampus of BD and controls. There are reports on the reduction of right hemisphere hippocampal volume in bipolar patients; some studies indicate that there is no change in the hippocampal size in BD compared to the control.[40–42] Yet, there is no uniform conclusion on the MRI changes in the hippocampus. The neurons are continuously exposed to endogenous oxidative stress, due to the brain’s high energy metabolism. The increased DNA fragmentation found in the thalamus, pons, frontal cortex, and medulla may be on account of the vulnerability to excitotoxicity? However, the intact DNA from the hippocampus may represent an adaptive compensation to oxidative stress. The genotoxic stress, the altered DNA topology, and also the insufficient DNA repair machinery may be the reason for the pathology of neurological disorders.[43] There are limited studies in the area of genomic biology of the brain and brain disorders. Yet, the mechanisms for neuronal cell death in brain disorders are not clear. It can possibly be due to both apoptotic and necrotic cell death events. Furthermore, the functional role of genomic stability in augmenting gene expression is still not clear. The DNA repair mechanisms are much more complex and the secrets of repair enzymes need to be further explored. The clinical and biological link between neuropsychiatry and neurodegeneration is overlapping, making it difficult for scientists and for drug discovery, as also for clinicians in diagnosis. There is a lot of debate on translational research and neurochemistry in overlapping clinical conditions in neuropsychiatry–neurodegenerative diseases. The major questions are, (i) Will MRI help to design the end-points to map the cross-talk between neuropsychiatry and neurodegeneration?, (ii) Does neurochemistry mapping help to interlink clinics and neurochemistry?, (iii) Will depression be an early event and risk factor for neurodegeneration?. There are three major challenges, (i) Can neuropsychiatry illness be a risk factor for neurodegeneration?, (ii) To map neurochemical and clinical complexities, to classify neuropsychiatry versus neurodegeneration, (iii) To develop biomarker end-points to classify neuropsychiatric and neurodegenerative disorders in independent and overlapping situations. A lot of debate on the topic of understanding the translational parameters related to diagnosis and drug discovery and also understanding the complex biology in overlapping clinical conditions like the neuropsychiatry–neurodegenerative features is still intense. We propose that failure in DNA repair due to altered DNA topology may be the early onset for the pathophysiology of brain disorders. This topic needs to be investigated further.

In conclusion the present study provides new evidence of the genome integrity in terms of DNA damage, stability, and conformation to ten different brain regions of BD compared to the age-matched controls, but whether this plays a central pathogenic role in causing the disease or is a consequence of the disease needs to be further investigated.

## References

[1] Nöthen MM, Nieratschker V, Cichon S, Rietschel M (2010). New findings in the genetics of major psychoses. Dialogues Clin Neurosci.

[2] Novick DM, Swartz HA, Frank E (2010). Suicide attempts in bipolar I and bipolar II disorder: a review and meta-analysis of the evidence Bipolar Disord.

[3] Brambilla, P, Glahn DC, Balestrieri M, Soares JC (2005). Magnetic resonance findings in bipolar disorder. Psychiatr Clin N Am.

[4] Rajkowska G, Miguel-Hidalgo JJ (2007). Gliogenesis and glial pathology in depression. CNS Neurol Disord Drug Targets.

[5] Benes FM, Vincent SL, Todtenkopf M (2001). The density of pyramidal and non pyramidal neurons in anterior cingulate cortex of schizophrenic and bipolar subjects. Biol Psychiatry.

[6] Benes FM, Kwok EW, Vincent SL, Todtenkopf MS (1998). A reduction of non pyramidal cells in sector CA2 of schizophrenics and manic depressive. Biol Psychiatry.

[7] Jou SH, Chiu NY, Liu CS (2009). Mitochondrial dysfunction and psychiatric disorders. Chang Gung Med J.

[8] Ng F, Berk M, Dean O, Bush AI (2008). Oxidative stress in psychiatric disorders: evidence base and therapeutic implicationss Int J Neuropsychopharmacol.

[9] Ranjekar PK, Hinge A, Hegde MV, Ghate M, Kale A, Sitasawad S (2003). Decreased antioxidant enzymes and membrane essential polyunsaturated fatty acids in schizophrenic and bipolar mood disorder patients. Psychiatry Res.

[10] Frey BN, Valvassori SS, Gomes KM, Martins MR, Pizzol DF, Kapczinski F (2006). Increased oxidative stress in submitochondrial particles after chronic amphetamine exposure. Brain Res.

[11] Frey BN, Andreazza AC, Kunz M, Gomes KM, Quevedo J, Salvador M (2007). Increased oxidative stress and DNA damage in bipolar disorder: A twin-case report. Prog Neuropsychopharmacol Biol Psychiatry.

[12] Benes FM, Matzilevich D, Burke RE, Walsh J (2006). The expression of proapoptosis genes is increased in bipolar disorder, but not in schizophrenia. Mol Psychiat.

[13] Chuang DM (2005). The antiapoptotic actions of mood stabilizers: molecular mechanisms and therapeutic potentials. Ann N Y Acad Sci.

[14] Catts VS, Catts SV (2000). Apoptosis and schizophrenia: is the tumour suppressor gene, p53, a candidate susceptibility gene?. Schizophr Res.

[15] Evan G, Littlewood T (1998). A matter of life and cell death. Science.

[16] Ansari B, Coates PJ, Greenstein BD, Hall PA (1993). In situ end-labelling detects DNA strand breaks in apoptosis and other physiological and pathological states. J Pathol.

[17] Benes FM, Walsh J, Bhattacharyya S, Sheth A, Berretta S (2003). DNA fragmentation decreased in schizophrenia but not bipolar disorder. Arch Gen Psychiatry.

[18] Buttner N, Bhattacharyya S, Walsh J, Benes FM (2007). DNA fragmentation is increased in non-GABAergic neurons in bipolar disorder but not in schizophrenia. Schizophr Res.

[19] Andreazza AC, Frey BN, Erdtmann B, Salvador M, Rombaldi F, Santin A (2007). DNA damage in bipolar disorder. Psychiatry Res.

[20] Alam ZI, Jenner A, Daniel SE, Lees AJ, Cairns N, Marsden CD (1997). Oxidative DNA damage in the parkinsonian brain: an apparent selective increase in 8-hydroxyguanine levels in substantia nigra. J Neurochem.

[21] Bonda DJ, Wang X, Perry G, Smith MA, Zhu X (2010). Mitochondrial dynamics in Alzheimer’s disease: opportunities for future treatment strategies. Drugs Aging.

[22] Hegde ML, Gupta VB, Anitha M, Harikrishna T, Shankar SK, Muthane U (2006). Studies on genomic DNA topology and stability in brain regions of Parkinson’s disease. Arch Biochem Biophys.

[23] Anitha S, Rao KSJ, Latha KS, Viswamitra MA (2002). First evidence to show the topological change of DNA from B-DNA to Z-DNA conformation from hippocampus of Alzheimer’s brain. J Neuromol Med.

[24] Sambrook J, Fritsch EF, Maniatis T (1989). Molecular Cloning-A Laboratory Manual.

[25] Sutherland BM (1983). Titration of pyrimidine dimer contents of nonradioactive deoxyribonucleic acid by electrophoresis in alkaline agarose gels. Biochemistry.

[26] Bhaskar MS, Rao KS (1994). Altered conformation and increased strand breaks in neuronal and astroglial DNA of aging rat brain. Biochem Mol Biol Int.

[27] Deng G, Wu R (1983). Terminal transferase: use of the tailing of DNA and for *in vitro* mutagenesis. Methods Enzymol.

[28] Chatterjee B, Rao GR (1994). Superhelical density of goat mitochondrial DNA: fluorimetric studies. Indian J Biochem Biophys.

[29] Gray M, Dratliff RL, Vaughan MR (1992). Circular Dichroism Spectroscopy of DNA. Methods Enzymol.

[30] Rao KS (1993). Genomic damage and its repair in young and aging brain. Mol Neurobiol.

[31] DelBello MP, Strakowski SM, Zimmerman ME, Hawkins JM, Sax KW (1999). MRI analysis of the cerebellum in bipolar disorder: a pilot study. Neuropsychopharmacology.

[32] Dasari M, Friedman L, Jesberger J, Stuve TA, Findling RL, Swales TP (1999). A magnetic imaging study of thalamic area in adolescent patients with either schizophrenia or bipolar disorder as compared to healthy controls. Psychiatry Res.

[33] Coffman JA, Bornstein RA, Olson SC, Schwarzkopf SB, Nasrallah HA (1990). Cognitive impairment and cerebral structure by MRI in bipolar disorder. Biol Psychiatry.

[34] Agarwal N, Port JD, Bazzocchi M, Renshaw PF (2010). Update on the use of MR for assessment and diagnosis of psychiatric diseases. Radiology.

[35] Adler CM, Holland SK, Schmithorst V, Wilke M, Weiss KL, Pan H (2004). Abnormal frontal white matter tracts in bipolar disorder: a diffusion tensor imaging study. Bipolar Disord.

[36] Kato T, Stine OC, McMahon FJ, Crowe RR (1997). Increased levels of a mitochondrial DNA deletion in the brain of patients with bipolar disorder. Biol Psychiatry.

[37] Rezin GT, Amboni G, Zugno AI, Quevedo J, Streck EL (2009). Mitochondrial dysfunction and psychiatric disorders. Neurochem Res.

[38] Mustak MS, Rao TSS, Shanmugavelu P, Sunder NM, Menon RB, Rao RV (2008). Assessment of serum macro and trace element homeostasis and the complexity of inter-element relations in bipolar mood disorders. Clin Chim Acta.

[39] Butters MA, Klunk WE, Mathis CA, Prince JC, Ziolko SK, Hoge JA (2008). Imaging Alzheimer Pathology in Late-life Depression with PET and Pittsburgh Compound-B. Alzheimer Dis Assoc Disord.

[40] Houser P, Dauphinais ID, Berrettini W, DeLisi LE, Gelemter J, Post RM (1989). Corpus callosum dimensions measured by magnetic resonance imaging in bipolar affective disorder and schizophrenia. Biol Psychiatry.

[41] Altshuler LL, Bartzokis G, Grieder T, Curran J, Mintz J (1998). Amygdala enlargement in bipolar disorder and hippocampal reduction in schizophrenia: an MRI study demonstrating neuroanatomic specificity. Arch Gen Psychiatry.

[42] Brambilla P, Nicoletti MA, Sassi RB, Mallinger AG, Frank E, Kupfer DJ (2003). Magnetic resonance imaging study of corpus callosum abnormalities in patients with bipolar disorder. Biol Psychiatry.

[43] Davydov V, Hansen LA, Shackelford DA (2003). Is DNA repair compromised in Alzheimer’s disease?. Neurobiol Aging.

